# Unraveling the Evolution of the Atlantic Cod’s (*Gadus morhua* L.) Alternative Immune Strategy

**DOI:** 10.1371/journal.pone.0074004

**Published:** 2013-09-03

**Authors:** Martin Malmstrøm, Sissel Jentoft, Tone F. Gregers, Kjetill S. Jakobsen

**Affiliations:** 1 CEES (Centre for Ecological and Evolutionary Synthesis), Department of Biosciences, University of Oslo, Oslo, Norway; 2 CIR (Centre for Immune Regulation), Department of Biosciences, University of Oslo, Oslo, Norway; University of London, St George’s, United Kingdom

## Abstract

Genes encoding the major histocompatibility complex (MHC) have been thought to play a vital role in the adaptive immune system in all vertebrates. The discovery that Atlantic cod (*Gadus morhua*) has lost important components of the MHC II pathway, accompanied by an unusually high number of MHC I genes, shed new light on the evolution and plasticity of the immune system of teleosts as well as in higher vertebrates. The overall aim of this study was to further investigate the highly expanded repertoire of MHC I genes using a cDNA approach to obtain sequence information of both the binding domains and the sorting signaling potential in the cytoplasmic tail. Here we report a novel combination of two endosomal sorting motifs, one tyrosine-based associated with exogenous peptide presentation by cross-presenting MHCI molecules, and one dileucine-based associated with normal MHC II functionality. The two signal motifs were identified in the cytoplasmic tail in a subset of the genes. This indicates that these genes have evolved MHC II-like functionality, allowing a more versatile use of MHC I through cross-presentation. Such an alternative immune strategy may have arisen through adaptive radiation and acquisition of new gene function as a response to changes in the habitat of its ancestral lineage.

## Introduction

The antigen presenting class I and II genes of the major histocompatibility complex (MHC) have been identified as crucial components of the adaptive immune system (AIS) in all higher vertebrates, including teleosts [1], [2]. Until now, it was generally believed that the MHC genes and their associated immune components have been conserved since their emergence in the jawed fishes, approximately 500 million years BP [1]–[3]. Sequencing of the Atlantic cod (*Gadus morhua*) genome [4], however, revealed the loss of MHC II as well as the MHC II interacting molecule CD4, required for T-cell activation, and the invariant chain (Ii), facilitating MHC II assembly, transport and peptide loading [5]. As an important part of the AIS, the antigen-presenting molecules MHC I and II help distinguish between self and non-self. Class I molecules normally present endogenously derived pathogens, typically of viral or tumoral origin, while class II molecules present exogenous pathogens such as bacteria and endoparasites. Post-infectional immunological memory, and the acquisition of immunity normally depend on the class II pathway [6]. Malfunction of the MHC II pathway is generally considered to lead to severe immune deficiency or even death.

Another unique feature of the immune system of Atlantic cod is the extreme expansion of MHC I genes. Earlier investigations have shown that Atlantic cod has an extended MHC I repertoire [7], [8], compared to other vertebrates. These findings were firmly established by the complete genome sequencing of this species, demonstrating that the Atlantic cod harbors about 100 copies of MHC I in its genome [4], more than twice as many as previously reported. In evolutionary time, the MHC I gene family has undergone several expansions, and subsequent reductions, especially following speciation and adaptive radiations within phylogenetic lineages [8], [9]. These differences are illustrated by the reduced MHC I and II repertoire of early Euteleosts [10], compared to the more advanced Neoteleost, like cichlids [11], [12]. The evolutionary arms race against co-evolving pathogens [13], co-evolution with commensal bacteria [14], and in some cases sexual selection [15], have all contributed to a diverse MHC repertoire. The extreme expansion of MHC I genes observed in cod, however, is unique in extant species, as most species retain a few highly conserved (yet polymorphic) “classical” (Ia), and several more divergent “non-classical” (Ib) MHC I genes [16]. It is currently unclear whether this expansion is functionally and evolutionarily linked to the loss of the MHC II pathway.

**Figure 1:**
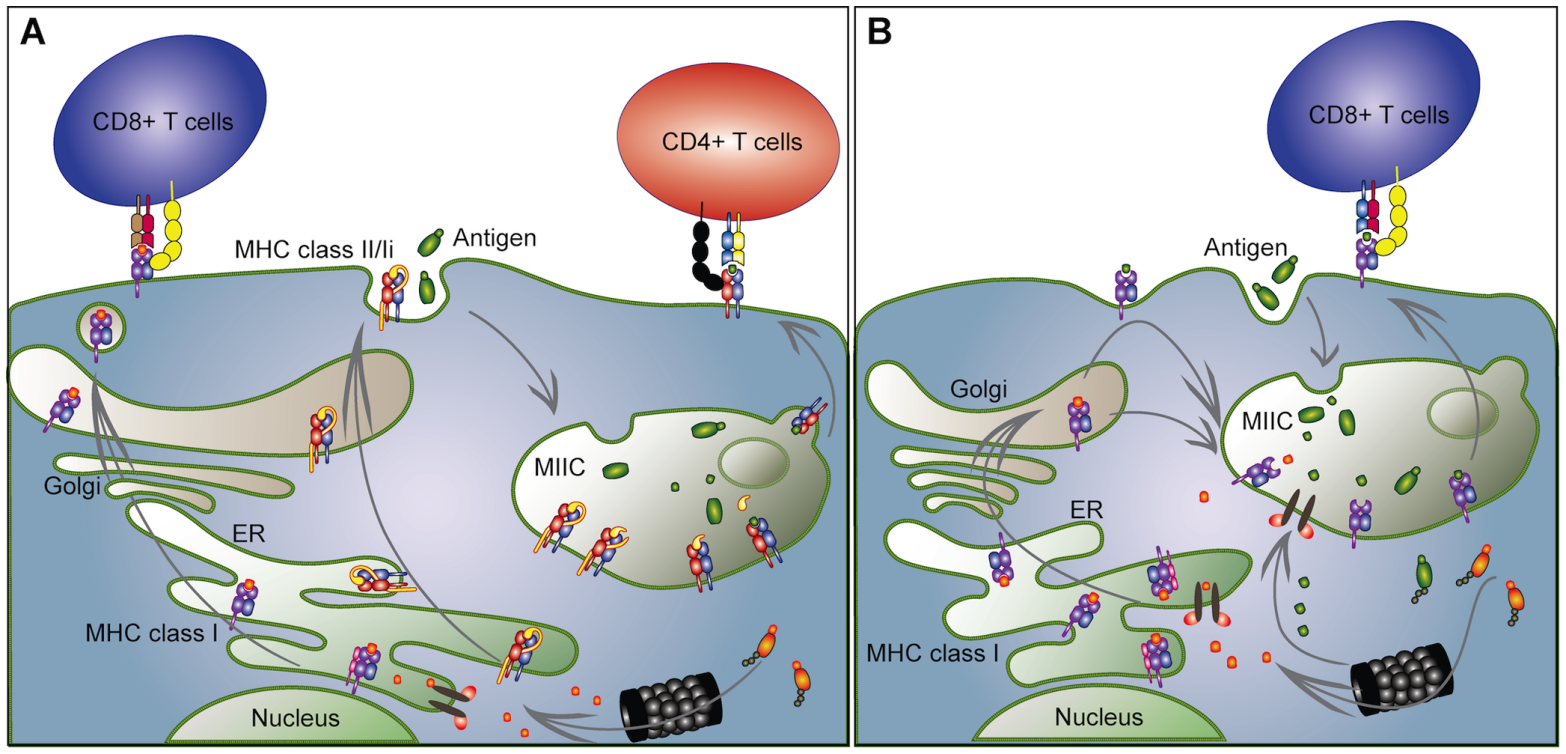
Classical and alternative pathways for antigen presentation. A) *Classical antigen presentation pathways*. MHC class I molecules assemble in the ER together with dedicated chaperones (like tapasin) that retain the MHC class I molecules until peptide binding. Ubiquitinated antigens are degraded by the proteasome, and the resulting peptides are transported via the transporters associated with antigen presentation (TAPs) into the ER lumen. Here the peptides are loaded onto MHC class I, tapasin is released and the peptide-MHC class I complex is transported through the Golgi to the cell surface where they are recognized by specific CD8+ T cells. MHC class II molecules also assemble in the ER with the dedicated chaperone Invariant chain (Ii). Ii mediates trafficking of MHC class II from the ER, through the Golgi, and via the cell surface to the endosomal pathway. Ii is exchanged for degraded exogenous antigenic peptides in specialized MHC class II loading compartments (MIIC). Peptide-loaded MHC class II molecules are released from the endosomal compartment to the cell surface where they are recognized by specific CD4+ T cells (reviewed in [6]. B) *Alternative (Cross-presentation) pathway for exogenous derived peptides by MHC I molecules*. MHC class I molecules carrying signal motifs in the cytoplasmic tail are transported to the endosomal pathway where endocytosed antigens are degraded. Peptides can then be loaded directly in the endosomes in a TAP-independent manner, or the antigens can translocate to the cytosol for proteasomal degradation. The processed antigens can then either be loaded on MHC class I in the ER, or transported back via TAP transporters that have been recruited to the endosomal membrane (reviewed in [35]). Peptide-loaded MHC class I molecules are subsequently released to the cell surface for antigen presentation to CD8+ T cells.

Furthermore, the immune functionality of Atlantic cod have caused some controversy due to contradicting reports on low to moderate specific antibody response [17], [18], and the fact that challenge tests show that Atlantic cod can survive, as well as establish immunity against bacteria [19], [20]. These findings indicate that components other than the classical adaptive immune system provide protection. One possible explanation is that the loss of MHC II functionality coincided with changes that allow a more versatile usage of MHC I, indicative through the expansion of this gene complex and the presence of two clades [4]. In mammals, it has been shown that CD8+ T cells can be activated through both the classical MHC I pathway as well as the alternative cross-presentation pathway [21], in which MHC I molecules mimic the function of class II molecules, presenting exogenous antigens to T-cells [22], [23]. In the classical MHC I pathway endogenously derived peptides are loaded onto MHC I within the ER and subsequently presented to CD8+ T-cells at the cell surface (Figure 1a) [24]. MHC II on the other hand is transported from the ER to the endosomal pathway facilitated by endosomal sorting signals within the cytoplasmic tail of the MHC II associated Ii. Here Ii is sequentially degraded and subsequently replaced by peptides derived from exogenous antigens taken up by the cell through endocytosis. Peptide-loaded MHC II is transported to the cell surface for presentation to CD4+ T cells (Figure 1a). In the cross-presentation pathway, exogenously derived peptides are presented in the context of MHC I, as phagocytized bacterial antigenic peptides are loaded onto phagosomal MHC I, which is recycled from the cell surface (Figure 1b) [21], [25], [26]. The relative importance and functionality of this alternative pathway in mammals is still debated [21], [27].

The cross-presentation pathway, like other intracellular transporting pathways, relies on trafficking of molecules facilitated by specific adaptor proteins which recognize and bind to intracellular sorting motifs embedded in the cytoplasmic tail of membrane spanning molecules [28], [29]. These conserved motifs act as signals, and in the AIS, they target proteins involved in pathogen recognition and transport them to the endosomes and lysosomes [30]. Dileucine-based and tyrosine-based motifs are the two main classes of sorting signals for endosomal trafficking, important in the degradation and preparation of extracellular antigen presentation. In humans, both signals have been shown to be involved in cross-presentation via MHC I [30], [31], while MHC II trafficking is exclusively facilitated by dileucine signals [32]. The functions of these signaling motifs are highly conserved in all vertebrates, including teleosts, and found in numerous membrane spanning molecules [33]. Both motifs are present in genes involved in antigen presentation in terrestrial vertebrates [30], whereas only dileucine-based signals have been reported in teleost MHC I [16] and II pathways [34].

The rationale for this study was to improve our understanding of the alternative immune system in Atlantic cod, by further characterization of the diverse repertoire of MHC class I genes. Of particular relevance was looking for the presence of sorting signals which would indicate enhanced cross-presentation functionality, thus allowing us to assess whether this pathway could have evolved to play a prominent role in the AIS. Here we report the discovery of a novel combination of sorting motifs in the cytoplasmic tail of MHC I molecules of Atlantic cod, and its proposed role in this alternative immune system.

## Results

### Expansion of MHC I Loci in Atlantic Cod

**Figure 2:**
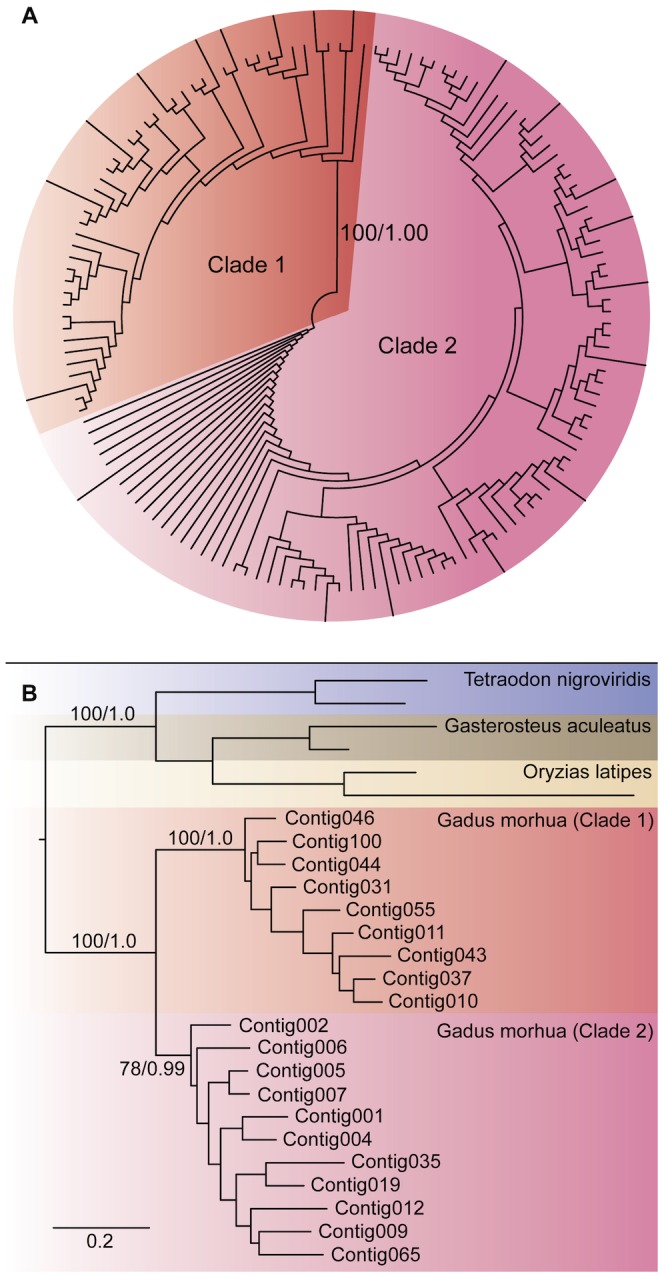
Phylogeny of MHC I diversity in Atlantic cod. A) Unrooted polar cladogram of all unique cDNA sequences of MHC Ia and Ib in Atlantic cod, based on amino acid sequence alignment. Elongated branches illustrate sequences originating from at least two independent PCR reactions. B) Subset of sequences highlighted in a), rooted with additional teleost Ia and Ib sequences from Ensembl. Maximum likelihood (ML) and Bayesian posterior probabilities are shown for the basal branches. Scale bar represents number of amino acid substitutions pr site.

In this study we investigated the complete coding regions of the transcribed MHC class I molecules, including the three α-domains, the transmembrane region, and the cytoplasmic tail. Numerous cDNA clones with correct insert length (≈ 1150 bp) were generated from 16 separate PCR reactions, and a total of 192 clones (12 per individual PCR reaction) were selected for Sanger sequencing (see Materials and Methods). Manual curation, including removal of duplicates and sequences likely containing PCR artifacts, reduced the number of unique nucleotide sequences to 143 (Figure 2a). Phylogenetic analysis confirms the previously observed split of these sequences into two fully supported main clades.

In order to link the information of binding abilities encoded by the α1 and α2 domains to any putative C-terminal signals, we focused our investigation on sequences we could confidently determine not to be chimeric due to PCR artifacts. Only sequences representing identical clones originating from two or more separate PCR reactions were included in the further analysis (see Materials and Methods). 20 sequences fulfilled this criterion (Figure 2b). The selected subset of sequences represents the majority of the basal branches observed in the complete dataset (elongated branches, Figure 2a).

### Structural Conservation of Sequences

**Figure 3:**
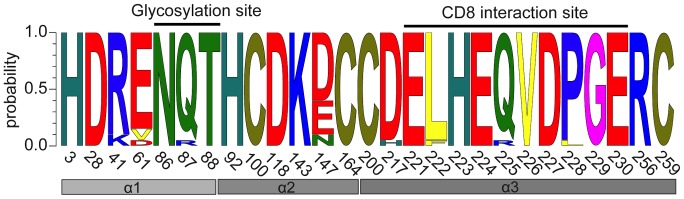
Conserved amino acid sites in typical MHC I molecules. WebLogo presentation of important selected structural and functional sites for subset of MHC I sequences from Atlantic cod. Letter size indicates the probability of the particular amino acids at the given site. Coloring scheme follows standard presentation in MEGA 5.05, reflecting amino acid properties. Numbering is based on consensus sequence, starting at the α1 domain (exon 2).

To determine whether the molecules encoded by either clade were atypical in any respect, we investigated the three-dimensional structure predicted by the sequence data. Several conserved features of typical MHC I structure and function were identified. The cysteine bridges in the alpha2 domain (pos. 100 and 164) and alpha3 domain (pos. 200 and 259) as well as the N-glycosylation site (**N**Q**T** at sites 86 to 88) are completely conserved in all sequences (Figure 3). Other important structural features, e.g. the conserved salt bridges, were also identified (H3-D28, R41-E61, H92-D118, K143-D/E147, D217-R256) in both clades. Further, the acidic domain presumed to be involved in recognition of the T-cell co-receptor CD8 (**E**LH**E**QV**D**PG**E** at pos. 221 to 230), was also present in all sequences. With the exception of Contig043, which apparently has no cytoplasmic domain, all sequences also contain a transmembrane region and a cytoplasmic tail consistent with typical MHC I structure and function.

### Sequence Variation in Binding Domains

In addition to overall structure, polymorphisms within the binding domains are an important trait in typical MHC class I molecules. Using the one-tailed Z-test for positive selection, we revealed a significant excess of non-synonymous mutations in the antigen presenting sites (APS) in both clades (Table 1, Figure 1). As these loci are paralogs from the same individual, signs of positive selection should be interpreted as balancing selection, and hence an evolutionary force promoting a diverse repertoire. Higher *d_N_* to *d_S_* ratio was also observed in non-APS in clade 2. There is no sign of positive selection in the highly conserved and structurally important exon 4 (α3 domain).

**Table 1:** Synonomus (*dS*) and non-synonomus (*dN*) mutations in functional sites of MHC I molecules.

		Selection	
Sites	no. sequences	*d_N_* –*d_S_*	*P*
Clade 1			
APS	9	**6,942**	**0,000**
non-APS	9	0,530	0,298
Exon 4	9	−0,885	1,000
Clade 2			
APS	11	**3,993**	**0,000**
non-ABS	11	**4,429**	**0,000**
Exon 4	11	0,269	0,616

Identification of antigen presenting sites (APS and non-APS) follows Kaufmann et. al. (1994), with 37 and 148 sites pr. sequence, respectively. Significant values in bold.

**Figure 4:**
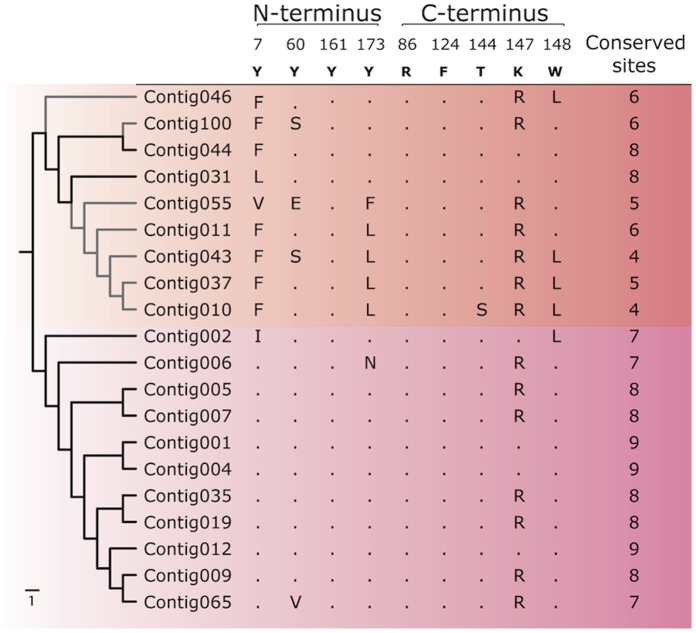
Anchoring sites in MHC I sequences. Amino acids found at conserved anchoring sites are shown for the selected subset of MHC Ia and Ib sequences in Atlantic cod. Conserved teleost amino acids are shown on top. Dots indicate coherence with conserved amino acid, while letters indicate substitute amino acids at each position for each contig. Gray branches represent Ib contigs containing six or fewer conserved sites. Numbering is based on consensus sequence, starting at the α1 domain (exon 2).

Based on the conserved anchoring sites in the binding groove (Figure 3) we assessed whether these molecules could potentially bind peptides. In clade 1, two sequences have sufficient conservation of anchoring sites to be regarded as classical (black branches in top half of Figure 3). Both sequences have eight of the nine anchoring sites conserved, indicating that these genes function as peptide-presenting molecules. The remaining clade 1 sequences are more divergent in their anchoring sites, where only four to six of these sites are conserved, indicating that these transcripts represent non-classical MHCI (Ib) (grey branches in Figure 4). In clade 2 all sequences are highly conserved in their anchoring sites, implying classical (Ia) function for this clade as a whole.

### Signaling Motifs in Cytoplasmic Tail

**Figure 5:**
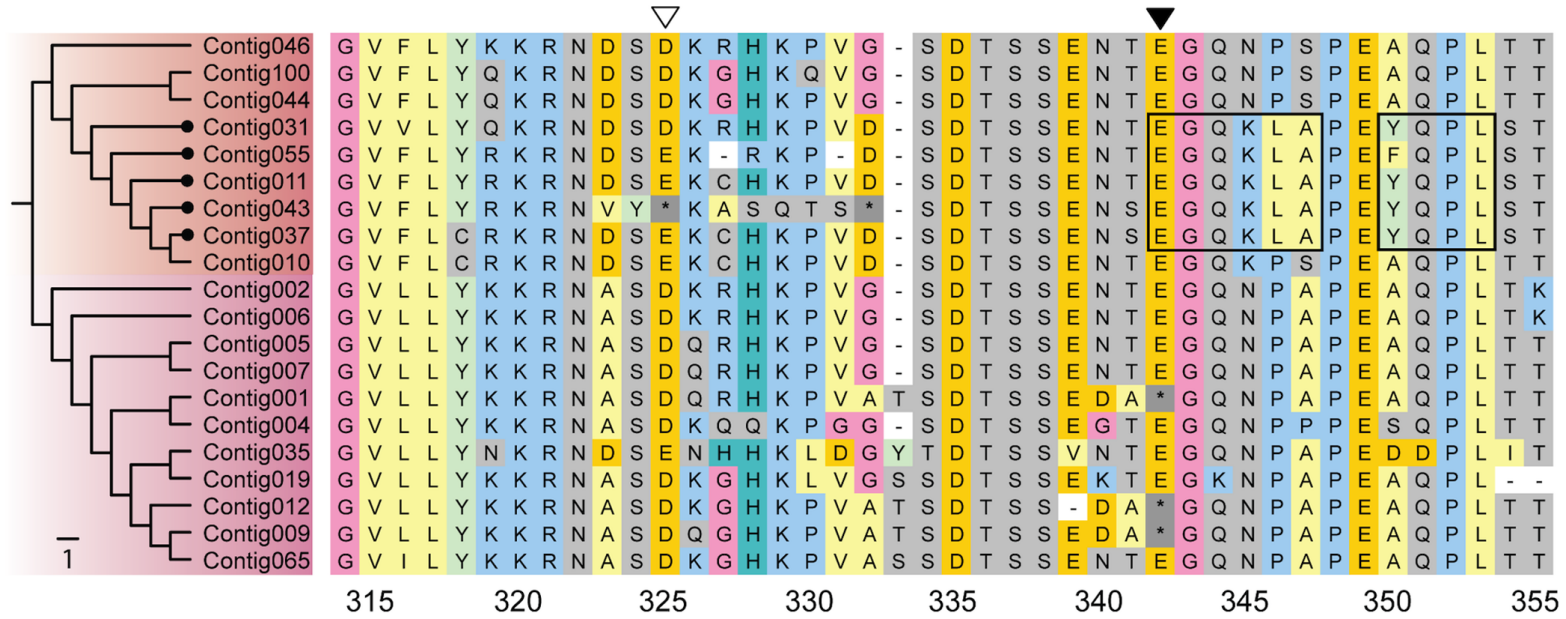
Signal motifs in cytoplasmic tail of MHC I. Amino acid sequences for a manually curated ClustalW nucleotide alignment for a selected subset of MHC Ia and Ib sequences in Atlantic cod. Targeting motifs are boxed (ExxxLA and YxxL), and sequences containing these are indicated with round terminal branches. Open and filled triangles indicate the position of a stop codon (*) in clade 1 and 2 respectively. The “−“ represent sequence gaps. The coloring scheme follows standard presentation in MEGA 5.05, reflecting amino acid properties. Numbering of amino acids is based on consensus sequence, starting at the α1 domain (exon 2).

Investigations of the cytoplasmic domain of the selected subset of sequences resulted in the discovery of two putative signal motifs for endosomal trafficking (Figure 5). These signals were only identified in sequences belonging to clade 1. The first signal is a dileucine-based motif (**E**GQK**LA**), found in five of the nine sequences in clade 1. The second motif is a tyrosine-based signal motif (**Y**QP**L**) located just two amino acids downstream of the first signal. In Contig055 the second signal contains a point mutation, where the tyrosine (Y) at position 350 has changed to phenylalanine (F), but given the chemical similarity between Y and F, this amino acid change is unlikely to be deleterious for the signal. The degree of nucleotide conservation surrounding the signal motifs suggests that both signals evolved through point mutations rather than by gene recombination mechanisms.

Stop codons causing premature termination were identified in both clades. In clade 1, an insertion leading to a stop codon at position 325 in Contig043 (open triangle in Figure 5) terminates the sequence following the transmembrane region, and thus eliminates the signal motifs during the process of amino acid translation. In clade 2, a point mutation (filled triangle in Figure 5), leads to a truncated cytoplasmic tail in three of the sequences.

Additionally, an extensive comparative analysis of all full-length MHC I coding regions available in the Ensembl Genome Browser from zebrafish, medaka, stickleback, tetraodon and tilapia (see Materials and Methods), revealed no sequences containing both motifs (File S1). A single putative tyrosine based motif was identified in tilapia, and several putative dileucine motifs were identified in a subset of the sequences in all species.

## Discussion

### Evolution of Novel Signaling Motifs in MHC I – Evidence for an Altered Immune Strategy

Signaling motifs are heavily involved in intracellular transport of immune related molecules. The type of motif determines what adaptor proteins they bind and regulates transportation of the molecule. Normally, one signal is sufficient as the molecules are specialized to follow one particular pathway, and conduct a specific task. Up until now, no molecule has been reported to carry two different signals.

Notably, in Atlantic cod the signal motifs are always found together, and only in one of the MHC I clades (clade 1), implying altered immune function for this clade. The signaling motifs resemble those known from the MHC II pathway [33], and cross-presentation pathway in other vertebrates [30]; this indicates that some of the clade 1 molecules have evolved to function more like class II molecules, as outlined in Figure 1b. This innovation may be an important part of the altered immune strategy that has evolved in Atlantic cod, enabling it to handle exogenous pathogens in absence of the normal MHC II pathway. The two signals will in theory allow the signal-carrying MHC I molecules to follow multiple trafficking pathways into the endosomal compartments and subsequently present extracellular peptides to T-cells [6], [35]. Sequences encoding Clade 2 molecules all appear to be classical (Ia) and without any signal motifs, thus they likely maintain the classical function of endogenous peptide presentation to CD8+ T-cells through the constitutive secretory route (see Figure 1a).

In what way the expansion of MHC I genes and the evolution of novel signaling motifs are linked to the loss of MHC II remains to be investigated. So far, two alternative ancestral selection scenarios have been suggested to explain the loss of MHC II in Atlantic cod [36]. One scenario explains the expansion of MHC I as a compensatory mechanism for the loss of the MHC II pathway, while in the other scenario the expansion occurred prior to the loss, rendering the MHC II system obsolete. Large-scale comparative genomics analysis of closely and distant related teleost lineages is needed to disentangle the two scenarios.

In mammalian systems MHC I and II genes are genetically linked [37], [38]. In teleosts however, this linkage is broken, as the gene clusters reside on different chromosomes [39]–[41]. This allows selection to act on each system independently. It follows that “alternative” (in comparison to mammalian) immune strategies are more likely to arise in the teleost lineage than in other vertebrate groups. The extreme expansion of MHC I genes, and the fact that these are divided into two well supported clades, suggests that these genes have been under strong positive (diversifying) selection, and indicates that they have evolved one or several novel functions within the Atlantic cod immune system. Our results suggest that the two clades have experienced different evolutionary pressures; one clade has maintained functionality reflecting ‘classical’ MHC I, while lack of evolutionary constraint has lead to MHC II-like functionality for some representatives of the other clade. As this alternative immune system may be shared with at least some of the other gadoids [4], this system is likely to have evolved millions of years ago.

### MHC Class Ia/Ib and MHC I-like Molecules

Both classical (Ia) and non-classical (Ib) MHC I molecules have the same typical appearance and organization, but Ib molecules have usually evolved to serve other immune-related functions such as lipid binding, NK-activation and other immune regulatory functions [42]. Some Ib molecules, however, have been shown to present bacterial antigens [43]. Ia loci are, by definition, highly polymorphic, but as data on population-based polymorphism of specific loci in these highly expanded genes is currently impossible to obtain for Atlantic cod, we have only used conserved anchoring sites in this study as an indication of Ia or Ib function. In this regard we find it valid to question whether the conventional definition of Ia and Ib function is applicable to the unconventional immune system we find in this species, as this definition is based on systems where both classes of MHC molecules are present [42].

The binding abilities are encoded in the groove constituted by the α1 and α2 domains [44]. Most of these antigen-presenting sites are polymorphic, but the nine most *N*- and *C*-terminal amino acids are highly conserved and function to anchor the peptide [45], [46]. In the 420my since teleosts diverged from their last common ancestor with mammals [47], the set of conserved mammalian anchoring sites (YYYYYYTKW) is somewhat different in the teleost lineage where the consensus is YYYYRTFKW [48]. In order to present peptides, MHC I molecules should have at least seven of these sites conserved, and thus be coined as classical (Ia) [49]. Interestingly, we find that most, but not all, of the sequences containing the signaling motifs have evolved towards non-classical function, and may no longer have the ability to present peptides. Of course, it should be considered whether the conserved set of anchoring sites for teleosts in general is strictly applicable for Atlantic cod. The prevalent replacement of lysine (K) with arginine (R) at position 147 in both clades seems to be specific for this species. Further analysis is needed to determine whether this arginine should actually be considered to be the most prevalent amino acid at this position. If so, additional clade 1 sequences would be coined classical. The fact that both variants (147 K/R) are found in both clades clearly indicates that the two clades originate from duplication of several genes, and not a single gene duplication event (see Figure 4). Nevertheless, some of the sequences in clade 1 seem to have evolved to serve other immune-related functions as they presumably have lost the ability to present peptides. In this regard, our results on Ia and Ib classification are consistent with findings in other teleosts such as medaka, cichlids, zebrafish, pufferfish, carp [50], rainbow trout [16] and salmon [51], as well as with previous investigations on Atlantic cod [8].

Our findings confirm the conserved structural characteristics of MHC I molecules (Figure 3), show a high degree of variability in the antigen presenting sites of the binding groove (Table 1) [52], and reveal an evolutionary pattern in the conserved anchoring sites (Figure 4) for both clades. Collectively these data support the notion that both clades originate from classical MHC I genes and do not represent MHC I-like molecules, or a separation purely of Ia and Ib sequences.

### Mutation Pattern in Anchoring Sites Indicate Early Evolution of Signaling Motifs

The mutation pattern of the anchoring sites in the binding groove follows the phylogeny of the complete transcripts to a great extent (see Figure 4). This correlation indicates that non-classical function has evolved several times within clade 1. All sequences in this clade have a mutation in the N-terminus, replacing tyrosine (Y) with a hydrophobic amino acid. This mutation most likely has only minor effects on the peptide binding ability, as the same mutation is also found at site 124(F) for all teleosts, compared to the mammalian counterpart [48]. Following the separation of the two clades our data show a mutation in the C-terminus where tryptophan (W) is replaced by leucine (L), presumably leading to non-classical function of the loci represented by Contig046. As the tryptophan (148 W) reappears in sequences branching off at the more distal nodes in the tree, some loci have retained the conserved amino acids – represented by contigs 044 and 031. This finding suggests that the evolution of the cytoplasmic signaling motifs has occurred prior to the emergence of genes represented by Contig031, indicating that these motifs most likely evolved basally in the Atlantic cod lineage.

## Conclusions

We here report the discovery of a novel combination of two sorting motifs that are normally associated with exogenous peptide presentation and cross-presentation by MHC class II molecules and MHC class I molecules, respectively. These findings indicate an altered functionality of MHC class I molecules in Atlantic cod and elucidate new insight into the plasticity and evolution of the vertebrate immune system.

## Materials and Methods

### Ethical Statement

We always aim to limit the effect of our research on populations and individuals. Whenever possible we collaborate with other sources, such as commercial fisheries or aquaculture farms, where samples can be harvested freely in combination with their normal business. This way, no animals need to be euthanized to serve our scientific purpose alone. The specimen used in this study comes from a wild population and was part of a larger haul of commercially fished individuals intended for human consumption. Following capture the fish were immediately stunned by bleeding following standard procedure by a local fisherman. Sampling in this manner does not fall under any specific legislation in Norway, but it is in accordance with the guidelines set by the ‘Norwegian consensus platform for replacement, reduction and refinement of animal experiments’ (www.norecopa.no).

### Sample Extraction and Purification

Spleen tissue from a single individual of Atlantic cod from the Lofoten area (68°8′48″N 13°36′35″E) in Norway was used. Total mRNA was extracted using ‘Dynabeads DIRECT mRNA Isolation Kit’ (Life Technologies, Carlsbad, California, U.S.), and gDNA was removed following the ‘Qiagen RNeasy MinElute Cleanup’ (Quiagen, Venlo, Netherlands) protocol. CDNA was synthesized using random hexamer primers (Roche, Penzberg, Germany) and ‘First strand cDNA Synthesis Kit’ (Fermentas, Vilnius, Lithuania). A final clean up and concentration was conducted with the ‘QIAquick PCR Purification Kit’ (Quiagen, Venlo, Netherlands), for a final concentration of 30 ul. All procedures were carried out following the manufacturers’ instructions.

### Amplification, Cloning and Sequencing

We chose to use cDNA in this experiment; this enabled us to sequence both the 5′ and 3′ end of the molecules and sequence them in one reaction. This was important as we wanted to investigate the cytoplasmic tail of these molecules, and couple any informational signals there to the upstream parts of the molecule. This approach also excludes any unexpressed pseudo-genes from the dataset. We have not attempted to analyze the total diversity of MHC class I, but rather illustrate the novelties which lay hidden in this diverse repertoire. Due to the extreme expansion and repetitive nature of the MHC I gene, with large regions present in near-identical copies between loci, it is still not possible, even with high throughput sequencing technologies and state-of-the-art bioinformatics, to assemble, classify and determine the genomic structure of all MCH I loci in Atlantic cod.

Universal MHC I primers for Atlantic cod, based on all available data from the Cod Genome Project (GenBank accession numbers JX567622 - JX567728) and other NCBI sequences (AJ132511–132529 and AF414203–AF414220), were designed for exon 1 (5′-CTGCTGTTGRTCTTTGGTCA) and exon 7 (5′-AAYGTGAGAAGMCTCTTCATG). As MHC I sequences are particularly prone to chimeric PCR generated errors [53], we ran 16 independent PCR reactions in parallel. Each PCR reaction of 10 µl was run with ‘BD Advantage 2 Polymerase Mix’ (BD Biosciences, San Jose, California, U.S.) under the following conditions: 94°C denaturation for 2 min, then running 25 cycles of 94°C 30 s, 56°C 30 s, 68°C 60 s, and 68°C elongation for 5 min. Following the PCR amplification, 3′-A- overhangs were added using ‘Dream-Taq DNA polymerase’ (Fermentas, Vilnius, Lithuania), before each pool of amplicons was cleaned up using ‘Wizard SV Gel and PCR Clean-Up System (Promega, Fitchburg, Wisconsin, U.S.). Cloning was performed independently for amplicons from each of the 16 PCR reactions, using ‘TOPO TA-Cloning Kit’ in ‘One Shot TOP10 Chemically Competent E. coli by Invitrogen (Life Technologies, Carlsbad, California, U.S.) following manufacturers instructions. 48 clones originating from each PCR reaction were screened on an agarose gel, of which 12 clones with the right insert size were picked, for a total of 192 clones. These were sequenced with conventional ABI 3730 technology. The 143 sequences included in this study have been submitted to GenBank with submission ID: 1563074.

### Sequence Handling and Phylogenetic Analysis

Raw sequence was manually inspected and corrected in Sequencher 5.0.1 (Gene Codes Corporation). Contigs representing unique sequences on a nucleotide level were aligned using ClustalW [54] as implemented in MEGA 5.05 [55] and the alignment was manually curated. Comparative sequences for rooting were downloaded from the Ensembl Genome Browser with accession numbers: ENSTNIT00000000248, ENSTNIT00000003613, ENSGACT00000002570, ENSGACT00000000184, ENSORLT00000021463 and ENSORLT00000008514; these are pufferfish (*Tetraodon nigroviridis*), stickleback (*Gasterosteus aculeatus*), and Medaka (*Oryzias latipes*), respectively.

Tree topology and bootstrapping (n = 100) for Maximum Likelihood were computed using RAxML HPC-PTHREADS (Version 7.2.6) [56] under the PROTGAMMAIJTTF model, suggested by ProtTest (Version 2.4) [57]. Bayesian posterior probabilities were calculated using MrBayes v3.1.2 [58], [59], run with 4 chains and with 5.0 million generations, and were sampled every 1000^th^ generation. Burnin was set to 40000. Site-specific rate model was set to “variable”, and the rate matrix for amino acids set to “fixed (jones)”. Parameters for the likelihood model were set to “invgamma”, and the model allowed the site-specific rate of change to vary over its evolutionary history using the “covarion” setting.

WebLogo was created using WebLogo 3 [60], and colours adjusted to MEGA 5.05 standard in Adobe Illustrator CS4.

### Comparative Analysis of Cytoplasmic Sequences

A total of 151 amino acid-translated transcripts from zebrafish, medaka, stickleback, tetraodon and tilapia were selected for comparative analysis of the cytoplasmic tail. These were detected using the in-built BLAST function of the Ensembl Genome Brower, with ENSDARP00000020667 (*Danio rerio*), ENSORLP00000001303 (*Oryzias latipes*), ENSGACP00000000148 (*Gasterosteus aculeatus*), ENSTNIP00000002995 (*Tetraodon nigroviridis*) and ENSONIP00000006183 (*Oreochromis niloticus*) as queries. All sequences were aligned using ClustalW [54] as implemented in MEGA 5.05 [55] individually for each species. Sequences too divergent to be aligned, or missing larger sections of sequence, including the cytoplasmic domain, were removed. The resulting 72 sequences were then manually inspected and compared to a selection of sequences from Atlantic cod, both with and without signaling motifs (File S1).

### Detection of Selection

Comparison of non-synonymous (dN) and synonymous (dS) mutations for detection of selection per site was done with the ‘One-tailed Z-test’ as implemented in MEGA 5.05 [55]. When the relative rate of dN to dS is equal (dN – dS = 0) a site is evolving neutrally. An excess of dN relative to dS (dN – dS >0) is indicative of positive (diversifying/balancing) selection, whereas an the opposite is indicative of purifying (negative) selection. The test report average values of dN-dS for each of the sequence partitions, and sequence sets tested. The P score represents the probability of rejecting the null hypothesis of strict-neutrality (dN = dS) in favor of the alternative hypothesis (dN>dS). The variance of the difference was estimated using the bootstrap method (1000 replicates). Analyses were conducted using the Nei-Gojobori method [61]. All positions containing alignment gaps and missing data were eliminated only in pairwise sequence comparisons (Pair-wise deletion option). A total of 285 positions were analyzed, 37 of which are defined as APS, 148 as non-APS, and 100 as Exon 4.

## Supporting Information

File S1 **Teleost cytoplasmic tail sequences.** Alignment of cytoplasmic tail for the 72 full-length MHC I coding regions available in the Ensembl Genome Browser from zebrafish (*Danio rerio*), medaka (*Oryzias latipes*), stickleback (*Gasterosteus aculeatus*), tetraodon (*Tetraodon nigroviridis*) and tilapia (*Oreochromis niloticus*). A subset of Atlantic cod (*Gadus morhua*) sequences, with and without signaling motifs, is included for comparison. All gaps have been removed.
(DOCX)
