# Correction: Unraveling the Evolution of the Atlantic Cod’s (*Gadus morhua* L.) Alternative Immune Strategy

**DOI:** 10.1371/annotation/18b70612-fd3d-46ce-a04b-652d18c82d5b

**Published:** 2013-10-25

**Authors:** Martin Malmstrøm, Sissel Jentoft, Tone F. Gregers, Kjetill S. Jakobsen

The last sentence of the "Amplification, Cloning and Sequencing" information was incorrect. The sentence should read:

"The 143 sequences included in this study have been submitted to GenBank with Accession numbers KF032934-KF033076." 

