# Supplementary material for: Unraveling the Evolution of the Atlantic Cod’s (Gadus morhua L.) Alternative Immune Strategy
Source: PLoS One. 2013 Sep 3;8(9):e74004. doi: 10.1371/journal.pone.0074004 (PMC3760826; doi:10.1371/journal.pone.0074004)
Supplement: File S1 — Teleost cytoplasmic tail sequences. Alignment of cytoplasmic tail for the 72 full-length MHC I coding regions available in the Ensembl Genome Browser from zebrafish (Danio rerio), medaka (Oryzias latipes), stickleback (Gasterosteus aculeatus), tetraodon (Tetraodon nigroviridis) and tilapia (Oreochromis niloticus). A subset of Atlantic cod (Gadus morhua) sequences, with and without signaling motifs, is included for comparison. All gaps have been removed. (DOCX) [file pone.0074004.s001.docx]

>Atlantic_cod_contig0100

GVFLYQKRNDSDKGHKQVGSDTSSENTEGQNPSPEAQPLTT

> Atlantic_cod_contig0044

GVFLYQKRNDSDKGHKPVGSDTSSENTEGQNPSPEAQPLTT

> Atlantic_cod_contig0031

GVVLYQKRNDSDKRHKPVDSDTSSENTEGQKLAPEYQPLST

> Atlantic_cod_contig0011

GVFLYRKKNDSEKCHKPVDSDTSSENTEGQKLAPEYQPLST

> Atlantic_cod_contig0037

GVFLCRKRNDSEKCHKPVDSDTSSENSEGQKLAPEYQPLST

> Atlantic_cod_contig0010

GVFLCRKRNDSEKCHKPVDSDTSSENTEGQKPSPEAQPLTT

>ENSORLP00000001201

LYCCWRNRNDVEMSPHGHHPHQMDPQPPGSLEVYKKQAIM

>ENSORLP00000008254

WLILYKRRKAADHLNKKKQILL

>ENSORLP00000008513

AFIFYKKKNAKRPPSPVDNKEIQEQMLQPENPSA

>ENSORLP00000008516

AFIFYKKKNVKIYLQ

>ENSORLP00000008515

AFIFYKKKNVSDLTAVDNKEIQEQMLQPENPSA

>ENSORLP00000008079

GSNMFKRLLNLQYCLCSSDKENKDVNGSICPQVDVSECN

>ENSORLP00000008539

AFIFYKKMNFFNLVKPPPSPVDNKEIQQQMLQPENPSA

>ENSORLP00000008541

AFIFYKKMNCKLL

>ENSORLP00000023398

LCCCWRNRNDENKKKKFLL

>ENSORLP00000015544

WLIVYKGRKVNIRLL

>ENSORLP00000023402

LCCCWRNRNVSDFQPVNGKCFLSSHLISFVFGTIDKQKVKCERR

>ENSORLP00000021462

IIVYYRKKAICPTLAKKEQNKSPKRQTRRPEDSSLPNT

>ENSORLP00000015536

WLIVYKGRKEFSQIKNKSGINDRTEKKKREEERGKLERG

>ENSORLP00000024359

LYCCWRNRNDFQPVDERFTRST

>ENSORLP00000015540

WLIVYKGRKDSFYIDFFAGPDGKEEEGRREREV

>ENSGACP00000000148

GFKVYRKRN

>ENSGACP00000000184

GFKLYRNRNAKPSSSSSASTEGSETSESGLVNI

>ENSGACP00000002383

GFKVYRKRNGER

>ENSGACP00000000198

GFKVHRKRNAKVQFGSVRQKDTESFTSAPQI

>ENSGACP00000000156

GFMVHRKRNAKCPS

>ENSGACP00000002561

GFKVYRKRNAKCSSAKCPSSSSSSSSTDGSDVTEELNPKPLKSAS

CTQRVAPRGHELFTAAVPQSCPQYEHKELLM

>ENSGACP00000002483

LSVWTGRESIHLGLKHPSTLIEISSIHTIFN

>ENSGACP00000002477

GFKVYRKRNAKCPSSTDGSEESLRDKLNPKP

>ENSGACP00000000197

GFKVHRKRNAKPSSSSSATTDGSDVTEELNPKP

>ENSGACP00000002519

GFKVHRKRNAQRSSAKPSSSSSASTDGSEVTEELNPKP

>ENSGACP00000002566

GFKLYRKRK

>ENSGACP00000000165

GFKVYRKRNGER

>ENSGACP00000000197(2)

GFKVHRKRNAKPSSSSSATTDGSDVTEELNPKP

>ENSGACP00000002491

GFKVYRNRNAKCP

>ENSGACP00000001674

SAKRSAAKSPFSSASTDGSDVTEELNPK

>ENSGACP00000001678

SAKRSAAKSPFSSASTDGSDVTEELNPKP

>ENSTNIP00000002995

GFLVYRQKKGKCPRDTPASDLGVELKLES

>ENSTNIP00000007774

GFIVYRQKKGKCPRAEGHPEVKEPLKPAA

>ENSTNIP00000000554

GFLVSRQRKGE

>ENSTNIP00000000273

GFLVYRQKVLAGKCPRAEGHPEVKEPLKPAPT

>ENSTNIP00000002167

GFIMYRQKKGESWR

>ENSONIP00000006183

GFTVYKKKKVSEQPPTSPPETDCELSERLNSETI

>ENSONIP00000004467

AFVIYKRKKGEKSYL

>ENSONIP00000008955

VFVTYKRKKGER

>ENSONIP00000008947

GFAVYKKKKVSERRSTSSPDNNAEQIQLNQVK

>ENSONIP00000004449

GFAVYKRKKGKQLKRLPCVNSPELEKILKHEVNCVH

>ENSONIP00000008959

MFVAYKRKKGERS

>ENSONIP00000008962

GVGVYKKKKERNTEPSLGDASEIQNLRCSYLSL

>ENSONIP00000008950

AVIAYKRKKGE

>ENSONIP00000008951

AFVAYKKKKGEKSECAPDDGSEQSERLNPQS

>ENSONIP00000008941

GFFIWKRNS

>ENSONIP00000004466

GFFVYKKKRGER

>ENSONIP00000010576

AFIIYKKRTDKRPPSPAENREVQEQMLPQA

>ENSONIP00000008927

GFFIWRRNSNEFQLANTGEQ

>ENSONIP00000004460

VVAAVVALSVAAVGFVVYKK

>ENSONIP00000010577

AFIIYKKRTGEKQ

>ENSONIP00000004459

LFILYWIPGGITAMLGITLDRE

>ENSONIP00000004454

IFSVAYKSNSNSSVSNTVFWTL

>ENSONIP00000015719

GFIIYKKKTVKLFNILYFSSSDSKSSSDGSSDI

>ENSONIP00000025183

TFLTYINKK

>ENSONIP00000015736

SFIVYKKRTGERKK

>ENSONIP00000025174

AFVTYKMKKGEKSECAPDDGSEQSERLNPQS

>ENSONIP00000025264

GVYMWQKKYKGFKPTNTSDTSST

>ENSONIP00000025178

AFVTYK

>ENSONIP00000023224

GFAVYKKKKGK

>ENSONIP00000023026

GIYFKLRGNGNEQNIFLCFMNDIHGLIVQTVNRRAVKSRQP

>ENSONIP00000025186

SEPKIMDLVCVFRTRSWESNNFKPKK

>ENSONIP00000025256

GIIVYKKKTSRCSQPPMKEPEVVKPLNPP

>ENSONIP00000025176

VFVTYKRKK

>ENSONIP00000025175

AFVTYKMKKGEKSE

>ENSONIP00000015737

SFIVYKKR

>ENSONIP00000015740

AFIIYKKRTGEK

>ENSONIP00000025270

GIFIWRRKYKGEK

>ENSDARP00000020667

GFVVYRRHKGFKPVPQNTSDGGSDNSSRT

>ENSDARP00000117273

GFVVYWKKKGFKPVPANPVNSDNDSGCGSGSDSVSHKSSDSTS

DISSVCSDNSSDSTTALLRNRQKYDKEN

>ENSDARP00000121274

GFVVYWKKKGFKPVPANPVNSDNDSGCGSGSDSVSHKS

>ENSDARP00000076471

CYLVYPKKLKPYQPVNQNEEMYEYIVKNE
